# Pinning and Anharmonic Phonon Effect of Quasi-Free-Standing Bilayer Epitaxial Graphene on SiC

**DOI:** 10.3390/nano12030346

**Published:** 2022-01-21

**Authors:** Li Sun, Peng Wang, Xuejian Xie, Xiufang Chen, Fapeng Yu, Yanlu Li, Xiangang Xu, Xian Zhao

**Affiliations:** State Key Laboratory of Crystal Materials, Center for Optics Research and Engineering, Shandong University, Jinan 250100, China; sdusunli@sdu.edu.cn (L.S.); wangpeng531@mail.sdu.edu.cn (P.W.); cxf@sdu.edu.cn (X.C.); fapengyu@sdu.edu.cn (F.Y.); liyanlu@sdu.edu.cn (Y.L.); xxu@sdu.edu.cn (X.X.)

**Keywords:** quasi-free-standing epitaxial graphene, H_2_ intercalation, evolution process

## Abstract

Epitaxial graphene on SiC without substrate interaction is viewed as one of the most promising two-dimensional (2D) materials in the microelectronics field. In this study, quasi-free-standing bilayer epitaxial graphene (QFSBEG) on SiC was fabricated by H_2_ intercalation under different time periods, and the temperature-dependent Raman spectra were recorded to evaluate the intrinsic structural difference generated by H_2_ time duration. The G peak thermal lineshift rates *dω/dT* showed that the H_2_ intercalation significantly weakened the pinning effect in epitaxial graphene. Furthermore, the G peak *dω/dT* value showed a perspicuous pinning effect disparity of QFSBEG samples. Additionally, the anharmonic phonon effect was investigated from the Raman lineshift of peaks. The physical mechanism responsible for dominating the G-mode temperature-dependent behavior among samples with different substrate coupling effects was elucidated. The phonon decay process of different samples was compared as the temperature increased. The evolution from in situ grown graphene to QFSBEG was determined. This study will expand the understanding of QFSBEG and pave a new way for its fabrication.

## 1. Introduction

Graphene grown on SiC has been viewed as the most promising method for graphene application because it can be used directly without the destructive transfer procedure [1,2,3]. Compared with C-terminated SiC, Si-terminated SiC substrate is widely used because the graphene is more uniform and the craft is more controllable. For the graphene grown on SiC (0001) face using commonly fabrication craft, a buffer layer exists between upper monolayer graphene and the SiC substrate underneath [4,5]. Among all kinds of atoms that can be intercalated between graphene and SiC substrate, like H_2_O, Li, Pb, etc. [6,7,8,9], H atom intercalation through the introduction of H_2_ into the growth furnace is the most widely used method to fabricate quasi-free-standing bilayer epitaxial graphene (QFSBEG), because H_2_ is compatible with the present gas circuit and friendly to subsequent device fabrication [10,11,12].

The difference between the structure and properties of in situ grown monolayer graphene and QFSBEG has been researched elsewhere [11,12,13,14]. After H intercalation, the Si-C bond between buffer layer and SiC substrate is broken. Hence, the original single-layer graphene with a buffer layer is turned into a bilayer graphene, which is viewed as quasi-free-standing bilayer epitaxial graphene (QFSBEG) [10,12]. Simultaneously, the electric properties of the device get promoted. For instance, the cutoff frequency ($f_{T}$) increased to 407 GHz [12], which is comparable to the recorded intrinsic graphene field effect transistor (GFET) at 427 GHz [15]. However, these properties were determined under a specific H_2_ intercalation condition, and how the evolution proceeds from the in situ graphene and QFSBEG have not been determined. The extent of H intercalation at the interface of the SiC substrate and the graphene has a direct influence on transport properties. Therefore, it is of vital importance to conduct a study on the progress from in situ graphene to QFSBEG.

For the characterization of QFSBEG, a mobility test is the most intuitive way to reveal the H_2_ intercalation condition using a Hall instrument. However, the sample can be damaged by the probe contact even under the simple four-probe Van der Pauw configuration. It is well known that Raman spectroscopy is one of the most effective and nondestructive methods for graphene characterization. In addition to the layer number and structure characteristics of graphene, the Raman spectra can also provide the anharmonic phonon effect by analyzing the temperature-dependent spectra. The anharmonic phonon effect is the collective effect of thermal expansion and electron-phonon and phonon-phonon interactions, which play an important role in electronic transport properties [16,17,18,19,20]. The temperature-dependent Raman spectra were recorded to disclose the evolution process and change in intrinsic physical properties from in situ graphene to QFSBEG.

In this study, the Raman spectra were measured as a function of temperature on in situ graphene and QFSBEG obtained under different H_2_ intercalation conditions in the temperature range from 213 K to 663 K. The substrate effect was compared through the line shift rates of G mode. Furthermore, the anharmonic phonon process was studied by conducting theoretical model calculations.

## 2. Materials and Methods

Epitaxial graphene was grown on home-made 4 inch SiC wafers in a self-developed furnace. The (0001) face was processed by chemical mechanical polishing (CMP) and followed by cleaning with acetone (manufactured by Yantai Yuandong Chemical Co. Ltd., Shandong, China), ethanol (manufactured by Tianjin Fuyu Fine Chemical Co. Ltd., Tianjin, China), and deionized water cleaning to remove surface contamination. The SiC substrate was subjected to a H_2_ etching procedure to obtain a regular and micron-scale step structure in a 5 sccm flow of H_2_ at a temperature of 1500–1600 °C and pressure of 800–900 mbar for 20 min. Graphene growth was conducted at 1600–1700 °C, 800–900 mbar in 20 sccm argon atmosphere for 60 min. The sample was then cooled to room temperature, corresponding to in situ graphene. For H_2_ intercalation, two samples were selected for H_2_ intercalation processing in a hydrogen flux of 50 sccm at a temperature of 1600 °C and pressure of 800–900 mbar. The operation time was selected as 30 min and 60 min to study the evolution process from in situ grown graphene to QFSBEG, corresponding to samples QFSBEG-1 and QFSBEG-2, respectively.

For the Raman measurement, HR 800 by Horiba Jobin Yvon (Paris, France) was used in the backscattering geometry with a 532 nm laser for excitation. For surface enhanced Raman spectra testing, the 100× objective was used with a 600 diffraction grating with the assistance of Ag particles at room temperature. For the temperature variation measurement, a Linkam (London, UK) hot/cold stage was used and cooled with liquid nitrogen. The 50× long-focus objective was used with a 600 diffraction grating restricted by the distance to the sample stage. The samples were measured from 213 K to 663 K in steps of 15 K. In the case of temperature interference on SiC substrate for substrate subtraction, a SiC substrate was placed in the hot/cold stage and its spectra were synchronously collected under different temperatures. Before each measurement, the samples were stabilized for about 5 min to ensure that the temperature had reached the set value and the optical system was refocused to avoid the thermal effect. For the accuracy of data, the positions of four samples (including a pure SiC substrate, in situ grown monolayer epitaxial graphene, QFSBEG-1, and QFSBEG-2) were recorded, and after each measurement, the stage was set at the same coordinate to ensure that the spectrum was taken at the same spot.

## 3. Results and Discussion

**Figure 1:**
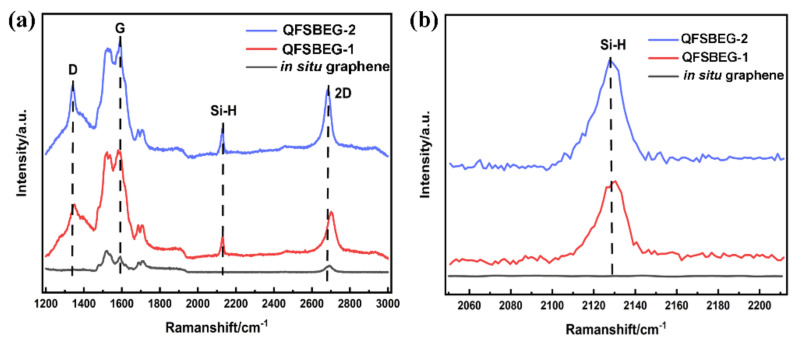
The whole surface enhanced Raman spectra (**a**) and Si-H peaks magnification (**b**) for in situ grown graphene, QFSBEG-1, and QFSBEG-2 using Ag nanoparticles.

The in situ grown graphene and the samples intercalated by H_2_ at different time periods were studied systematically. First, in order to get intuitive understanding of graphene after H_2_ intercalation, the surface enhanced Raman spectra of these three samples were measured with the assistance of Ag particles. as shown in Figure 1a. Compared with the in situ graphene, an extra peak at ~2130 cm^−1^ appeared in the Raman spectra of samples after H_2_ intercalation. It was considered that the perpendicular stretch mode of the Si-H bond was excited by the near-field plasmonic effect, thus, the H_2_ intercalation could be proved straightforward in comparison with the conventional method [13,21]. However, referring to the Si-H peaks as depicted in Figure 1b, there was almost no difference in the Raman spectra for samples QFSBEG-1 and QFSBEG-2. In consequence, the enhanced Raman spectra could only indicate whether the H_2_ was intercalated successfully or failed, but they did not provide information about the inner condition of QFSBEG.

**Figure 2:**
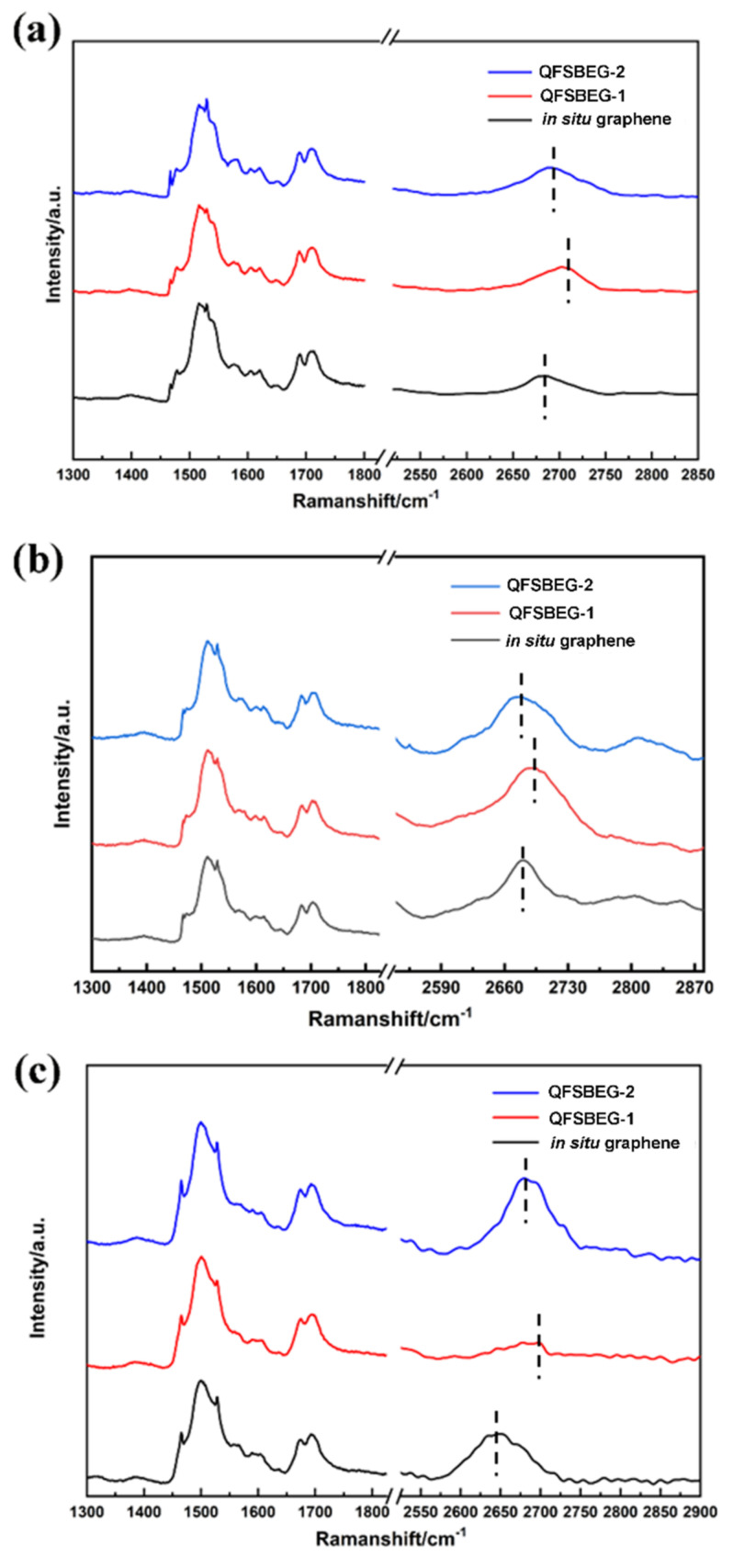
Typical Raman spectra of three samples at temperature of: (**a**) 273 K, (**b**) 423 K, and (**c**) 633 K, respectively.

Raman spectra of three samples were measured in the temperature range of 213K–663K, and the spectra obtained in three typical temperatures (273 K, 423 K, and 633 K) are shown in Figure 2. The G peak was not obvious due to the effect of a second-order peak of SiC substrate. As a result, the detailed information about G peak should be obtained by subtracting the SiC spectra and will be discussed below. For the two-dimensional (2D) peak, the spectra displayed the same tendency under different temperatures. In addition, the sample QFSBEG-2 displayed a blueshift compared with the in situ grown graphene on SiC, whereas it showed a redshift compared with QFSBEG-1 at a certain temperature. According to previous studies, the 2D peak position could reflect the strain between graphene and the SiC substrate [18,22]. The redshift of the 2D peak in the Raman spectrum proved the interaction between graphene and substrate for 60 min was less than that in 30 min.

**Figure 3:**
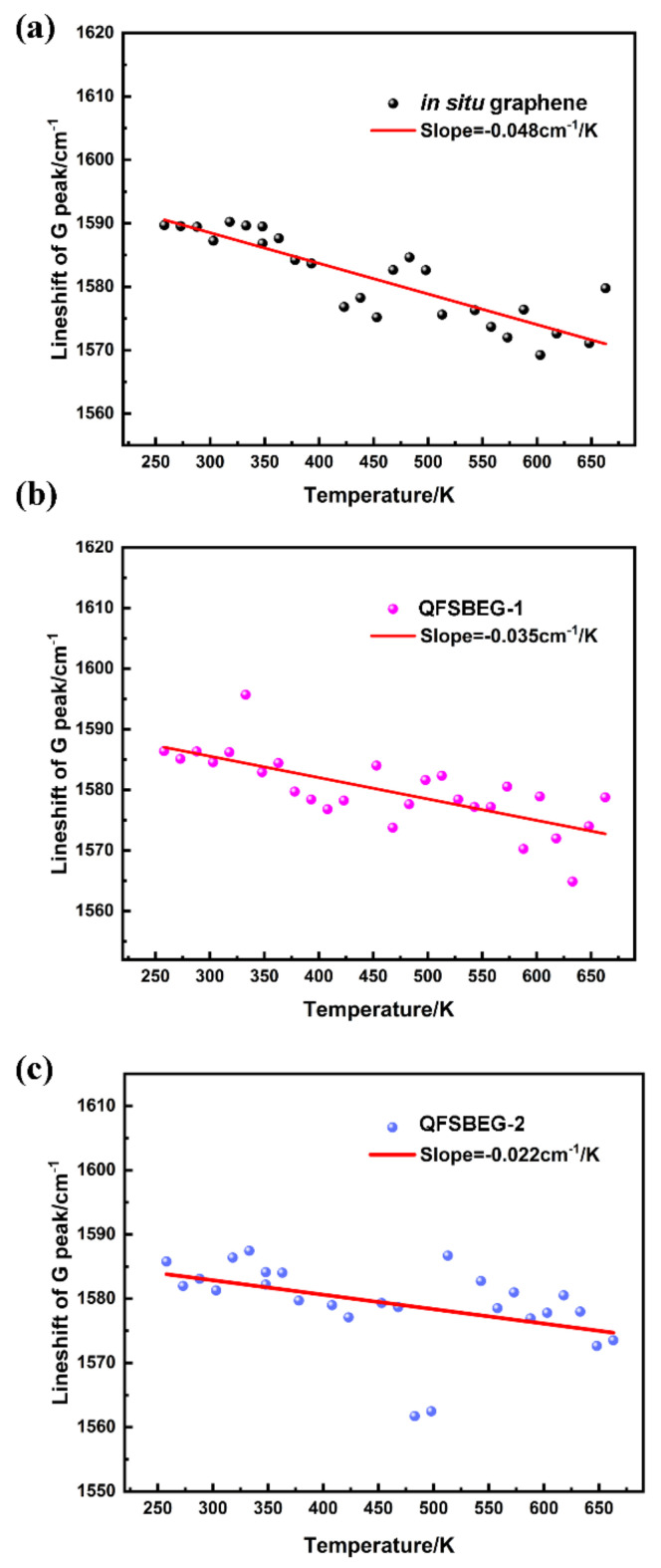
The lineshift of G peak as a function of temperature for in situ graphene (**a**), QFSBEG-1 (**b**), and QFSBEG-2 (**c**).

For the analysis of the G peak, the Raman spectra of graphene were subtracted by the SiC Raman spectra simultaneously taken at a specific temperature. The subtraction process was shown in Supplementary Materials. After the subtraction, the influence of second-order SiC peaks were avoided and the G peak become obvious. The G peak positions for these three samples under different temperatures are shown in Figure 3, and the thermal lineshift rates *dω/dT* are summarized in Table 1. Moreover, the rates for graphene on the other substrate are also listed in Table 1 for a perspicuous comparison. The thermal shift rate is a quantitative method for analyzing the pinning effect by the substrate [23,24]. The in situ grown graphene on the SiC sample in this study presented a *dω/dT* rate of −0.048 cm^−1^/K, which was consistent with the reported in situ grown epitaxial graphene on SiC rate of −0.043 cm^−1^/K [24]. After H_2_ intercalation, the value declined dramatically and varied by the time duration of H_2_ intercalation. The *dω/dT* rates of samples QFSBEG-1 and QFSBEG-2 were −0.035 cm^−1^/K and −0.022 cm^−1^/K, respectively. It is clear that the *dω/dT* rate is an effective parameter for characterizing the intrinsic state of the substrate pinning effect under different H_2_ conditions. As the time extended, the substrate effect would be further released but beyond the reach of free-standing graphene at the rate of ~−0.010 cm^−1^/K [25].

It is worth noting that, with the extent of H_2_ intercalation deepening from Figure 3a to Figure 3c, the dispersion of the G mode lineshift data increased, especially at temperatures higher than 350 K. The data divergence can be explained by two reasons. First, it can be accounted for by the stability of the H atom. At a high temperature, the H bond can be broken and the H atoms would escape from the SiC substrate. In addition, in consideration of the effect on the temperature-dependent Raman spectra, the lattice mismatch between the host atoms and doping atoms can contribute to the spectral variation, especially for samples that were epitaxial grown on SiC substrate.

For a typical Raman spectrum of graphene, it is of vital importance to study the G, D, and 2D peaks. The G peak is representative of *sp2* carbon hybridization and resulted from the degenerate phonon mode *E_2g_* at the center of Brillouin zone. The D peak is contributed by a one-phonon process assisted by the defect near the K point of the Brillouin zone, thus it is sensitive to the crystalline defects. The 2D peak is an overtone of the D peak, which originated from a second-order phonon process and is sensitive to the number of graphene layers. For a Raman spectrum taken at room temperature, the domain size or the layer of graphene could be extracted through these three peaks using an empirical equation. Temperature-dependent Raman spectra are an excellent tool to investigate anharmonic effects. According to the ab initio calculation, the Raman peak position is the real part of the phonon self-energy, and the Raman linewidth is the imaginary part of the self-energy. The Raman shift of G peak at a certain temperature can be expressed using the following formula [28,29].

(1) $$\Omega \left(T\right)=\Omega_{0}+\Delta^{\left(1\right)}\left(T\right)+\Delta^{\left(2\right)}\left(T\right)$$

where $\Omega_{0}$ is the harmonic frequency. The expression $\Delta^{\left(1\right)}\left(T\right)$ represents the line shift contributed by the thermal expansion and can be expressed as follows:

(2) $$\Delta^{\left(1\right)}\left(T\right)=\Omega_{0}\left\{exp\left[-\gamma \int_{0}^{T}\beta (T^{\prime })dT^{\prime }\right]-1\right\}$$

where *γ* is the Gruneisen parameter of graphene for the Raman G mode; here, we take *γ_G_* = 1.99 [30]; $\beta \left(T\right)$ is the coefficient of the volume thermal expansion of graphene; in this study, the thermal expansion coefficient of graphite $\alpha_{graphite}$ was used [31].

(3) $$\beta \left(T\right)=\alpha_{graphite}=3.46832*10^{-6}+1.73185*10^{-9}T-5.79967*10^{-13}{\;T}^{2}$$

Here, the simple Klemens model was used to clarify the Raman shift variation caused by the pure temperature effect. In this model, the effect can be considered as three-phonon and four-phonon interactions, and can be expressed as follows:

(4) $$\Delta^{\left(2\right)}\left(T\right)=M_{1}\left(1+\overset{2}{\underset{i=1}{\sum }}n\left(T,\omega_{i}\right)\right)+M_{2}\left(1+\overset{3}{\underset{j=1}{\sum }}n\left(T,\omega_{j}\right)+\overset{3}{\underset{j=1}{\sum }}n^{2}\left(T,\omega_{j}\right)\right)$$

where *M*_1_ and *M*_2_ are constants representing the three-phonon and four-phonon processes to the Raman shift, respectively. The term n(T,*w*) represents the Bose-Einstein function and can be described by the following equation:

(5) $$n\left(T,\omega \right)={\left(e^{ћw/k_{B}T}-1\right)}^{-1}$$

**Figure 4:**
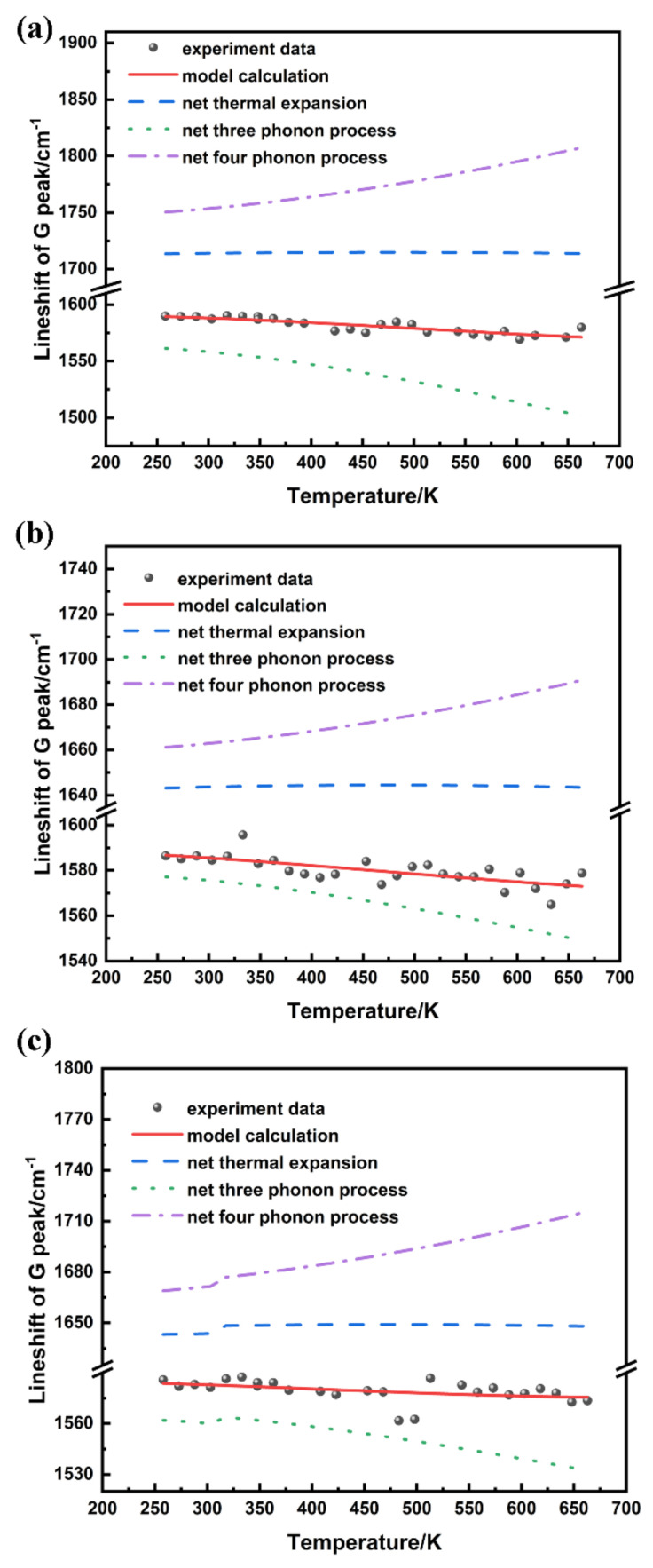
The experimental data and model calculation of G mode for in situ grown graphene (**a**), QFSBEG-1 (**b**), and QFSBEG-2 (**c**).

Figure 4 shows the temperature dependence of the G peak line shift of these three samples in the temperature range of 213–663 K. The black circles represent the experimental data and the red line represents the calculated result obtained by the model. The fitting parameters *Ω*_0_, *M*_1_, and *M*_2_ are shown in Table 2. Furthermore, the effect of net thermal expansion and net three and four phonon processes on the lineshift were also calculated in blue, green, and purple dash lines, respectively. The fitting curve matched well with the experimental data. The *Ω*_0_ of the QFSBEG samples exhibited a redshift, in contrast with the in situ graphene. In consideration of the phonon process, the reported studies showed that the four-phonon effect was the dominating factor over the thermal expansion and three-phonon process G mode with the temperature [26,32]. In contrast, a three-phonon process was the primary aspect in the graphene grown on the SiC substrate from data extracted in this study. Remarkably, studies related to anharmonic effect on SiC crystal showed that three-phonon process played a leading role [33]. Indeed, the intrinsic linewidth $\Omega^{in}$ in a defect-free sample can be attributed to the interactions of electron-phonon and anharmonic phonon-phonon. As shown in our previous work, both the in situ grown graphene and QFSBEG samples exhibited a coupling effect in the calculated band structure [34]. It can be deduced that in the epitaxial graphene, the phonon decay of graphene can be coupled with the SiC substrate. The M_1_/M_2_ value of QFSBEG-2 decreased in contrast with that of QFSBEG-1, which indicated that the three-phonon effect decreased with the extent of H intercalation deepening.

Usually, the phonon damping rate is proportional to the linewidth Γ(T) of the Raman peak. Hence, the phonon lifetime τ can be calculated from the peak linewidth Γ through the energy-time uncertainty relation, and the equation can be expressed as follows [34]:

(6) $$\frac{\Gamma }{ћ}=\frac{1}{\tau }$$

Here, ћ is the reduced Planck constant and ћ = 5.3 cm^−1^∙ps. Based on the G mode, the peak width was extracted by Lorentz fitting and the phonon lifetime was calculated by the above equation. The linewidth of the Raman G peak and phonon lifetime of these three samples are shown in the Table 3 in the temperature range of 273–423 K. From these data, it can be observed that the phonon lifetimes τ of samples were at the sub-picosecond magnitude and increased as the temperature went up. In general, the phonon lifetimes τ of in situ grown graphene were lower than those of both QFSBEG when the temperature was less than 333 K. As the temperature continued to increase, the phonon lifetime τ of in situ grown graphene prolonged significantly, whereas the conditions were different for QFSBEG-1 and QFSBEG-2. The phonon lifetime τ of QFSBEG-1 exhibited a slight variation and fluctuated at the temperature of 423 K. The phonon lifetime τ of QFSBEG-1 steadily increased but the range was smaller than that of in situ grown graphene. It is worth noting that the phonon lifetime τ of all samples began to descend and remained at a relatively low value of ~0.1 ps.

For the measured phonon lifetime *τ*, it was affected by two main factors, and the equation can be expressed as [35,36]:

(7) $$\frac{1}{\tau }=\frac{1}{\tau_{A}}+\frac{1}{\tau_{I}}$$

where $\tau_{A}$ and $\tau_{I}$ represent for the decay times caused by anharmonicity and the impurity scattering, respectively. To be specific, $\tau_{A}$ is intrinsically scattered by the anharmonicity of crystal lattice and is the dominant factor for the calculated τ. However, $\tau_{I}$ appears when there exists impurity or defect in the crystal, thus extra decay pathways are afforded for the phonon scattering. Because these three samples were all unintentionally doped, they can be viewed as intrinsic graphene. The value of phonon lifetime τ will be almost affected by that of $\tau_{A}$.

**Figure 5:**
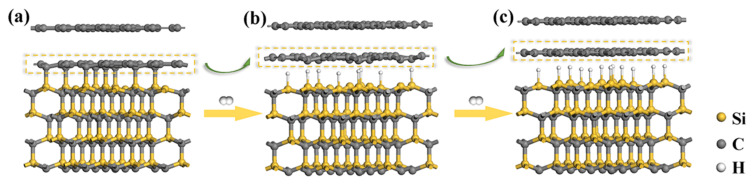
Schematic diagram of QFSEG transformation from in situ grown graphene (**a**) under different H_2_ duration time: (**b**) 30 min; and (**c**) 60 min.

Based on the experimental data, the transformation evolution process from in situ graphene to QFSBEG is summarized in Figure 5. Figure 5a was the calculated model after structure geometry optimization by the first principle theory. As shown in Figure 5a, the SiC substrate and first layer C atoms marked with the dashed box were bonded and the distance was shorter than that of upper C atoms layer. The substrate and buffer layer were strongly bonded and the pinning effect was very high. After 30 min of H_2_ processing, the Si-C bonds were broken and the Si dangling bonds were partially saturated by H atoms, as shown in Figure 5b. Nevertheless, the first C atoms layer was still wrinkled due to the mutual pinning effect still being higher than that of free-standing graphene. As the time continued to extend, more H atoms were intercalated between the first C atoms layer and the SiC substrate in Figure 5c. The pinning effect got further released and the first C atoms layer became flat and became the real sense of graphene.

## 4. Conclusions

In conclusion, in situ grown graphene on SiC and QFSBEG under different H_2_ intercalation time periods were fabricated. Temperature-dependent Raman spectra were collected in the temperature range of 213–663 K. In contrast to the in situ grown graphene that the G peak *dω/dT* value at changed at the rate −0.048 cm^−1^/K, the G peak thermal lineshift rates *dω/dT* declined to −0.035 cm^−1^/K after 30 min H_2_ management and −0.022cm^−1^/K after 60 min H_2_ management, respectively. The anharmonic effect analysis showed that the three-phonon process was the dominant decay pathway, and the ratio of the three-phonon process to the four-phonon process dropped from 4.74 to 3.69 as the H_2_ treatment time increased. At the conclusion, the evolution process of transformation was demonstrated.

## Figures and Tables

**Table 1:** Thermal lineshift rate comparisons for graphene fabricated under different conditions.

Sample	*dω/dT* (cm^−1^/K)	T Range (K)	Theory
Freestanding [25,26]	−0.009 ± 0.002	150–250	−0.011
	−0.015 ± 0.003	300–400	−0.017
Pressed on SiO_2_/Si [24]	−0.052 ± 0.004	300–400	−0.046
On Au/SiN/Si [27]	−0.040 ± 0.002	400–500	−0.052
In situ grown epitaxial graphene [24]	−0.043 ± 0.013	300–400	−0.048
In situ grown epitaxial graphene in this study	−0.048 ± 0.005	258–663	
QFSBEG-1 in this study	−0.035 ± 0.006	258–663	
QFSBEG-2 in this study	−0.022 ± 0.008	258–663	

**Table 2:** The G mode fitting parameters of Raman spectra for different graphene samples using the fitting model.

	In Situ Graphene	QFSBEG-1	QFSBEG-2
Ω_0_ (cm^−1^)	1713.68	1643.37	1647.93
M1	−149.01	−64.50	−79.20
M2	31.43	14.95	21.44
M1/M2	4.74	4.31	3.69

**Table 3:** Linewidth Γ of Raman G peak (cm^−1^) and phonon lifetime *τ* (picosecond) of phonon mode for different samples at variable temperature.

Temperature	In Situ Graphene		QFSBEG-1		QFSBEG-2	
	Γ (cm^−1^)	*τ* (ps)	Γ (cm^−1^)	*τ* (ps)	Γ (cm^−1^)	*τ* (ps)
273 K	18.557	0.286	13.099	0.405	16.036	0.331
303 K	19.969	0.265	15.549	0.341	11.576	0.458
333 K	28.387	0.187	16.756	0.316	10.724	0.494
363 K	9.075	0.584	15.888	0.334	11.751	0.451
393 K	4.788	1.107	11.803	0.449	8.007	0.662
423 K	4.348	1.219	30.242	0.175	7.520	0.705

## Data Availability

The data presented in this study are available on request from the corresponding authors.

## Supplementary Materials

The following supporting information can be downloaded at: https://www.mdpi.com/article/10.3390/nano12030346/s1, Figure S1: Raman spectra of pure SiC substrate (a), graphene before (b) and after (c), (d) subtraction.
