# Supplementary material for: Pinning and Anharmonic Phonon Effect of Quasi-Free-Standing Bilayer Epitaxial Graphene on SiC
Source: Nanomaterials (Basel). 2022 Jan 21;12(3):346. doi: 10.3390/nano12030346 (PMC8839960; doi:10.3390/nano12030346)
Supplement: Supplementary file 1 [file nanomaterials-12-00346-s001.zip › nanomaterials-1499875-supplementary.pdf]

nanomaterials

MDPI

Supplementary Materials

Pinning and Anharmonic Phonon Effect of Quasi-Free-Standing Bilayer Epitaxial Graphene on SiC

Li Sun, Peng Wang, Xuejian Xie *, Xiufang Chen, Fapeng Yu, Yanlu Li, Xiangang Xu and Xian Zhao *

State Key Laboratory of Crystal Materials, Center for Optics Research and Engineering, Shandong University, Jinan 250100, China; sdusunli@sdu.edu.cn (L.S.); wangpeng531@mail.sdu.edu.cn (P.W.); cxf@sdu.edu.cn (X.C.); fapengyu@sdu.edu.cn (F.Y.); liyanlu@sdu.edu.cn (Y.L.); xxu@sdu.edu.cn (X.X.)

* Correspondence: xiexj@sdu.edu.cn (X.X.); xianzhao@sdu.edu.cn (X.Z.)

The directly obtained spectra of the epitaxial graphene was shown in Fig. S1b. G peak was not obvious due to the effect of second-order peak of SiC substrate. The detailed information about G peak should be obtained by subtracting the simultaneously SiC spectra at the same temperature. After the substrate subtraction in Fig. S1c, the influence of second-order SiC peaks were avoided and the G peak become obvious. The Fig. S1c owns the same Y scale with that of Fig. S1a and Fig. S1b. Usually, the spectrum will be enlarged in the magnification as shown in Fig. S1d for the convenience of observation.

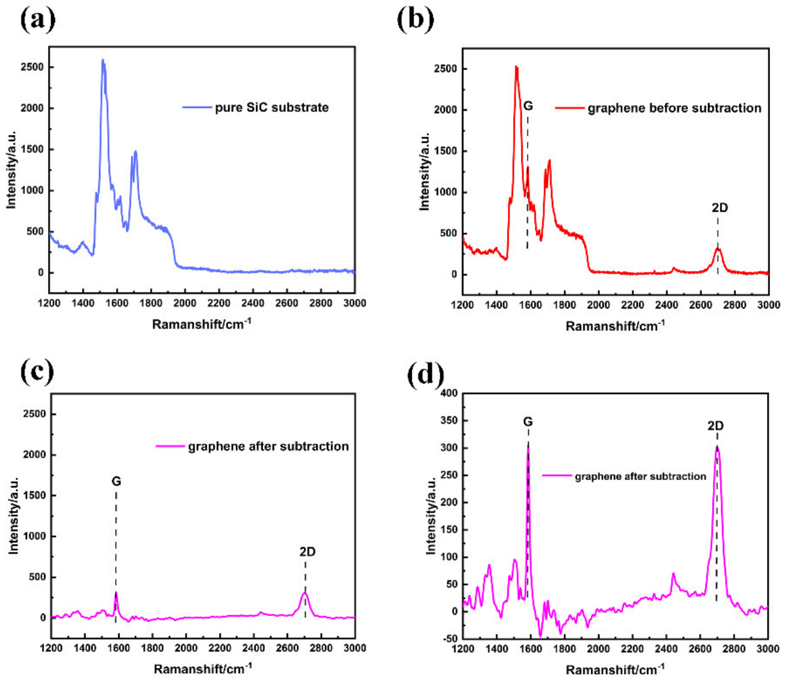

Figure S1. Raman spectra of pure SiC substrate (**a**), graphene before (**b**) and after (**c**), (**d**) subtraction.
